# Acetosyringone treatment duration affects large T-DNA molecule transfer to rice callus

**DOI:** 10.1186/s12896-018-0459-5

**Published:** 2018-08-09

**Authors:** Jing Xi, Minesh Patel, Shujie Dong, Qiudeng Que, Rongda Qu

**Affiliations:** 1 Department of Crop and Soil Sciences, North Carolina State University, Raleigh, NC 27695 USA; 2 Present address: Department of Biochemistry, University of California, Riverside, CA 92521 USA; 3 Present address: BASF Corporation-R&D Center, Durham, NC 27709 USA; 4 Syngenta Crop Protection, LLC, 9 Davis Drive, Research Triangle Park, Durham, NC 27709 USA

**Keywords:** *Agrobacterium*, BIBAC, Immuno-precipitation, Large T-DNA, Monocot transformation

## Abstract

**Background:**

Large T-DNA fragment transfer has long been a problem for *Agrobacterium*-mediated transformation. Although vector systems, such as the BIBAC series, were successfully developed for the purpose, low transformation efficiencies were consistently observed.

**Results:**

To gain insights of this problem in monocot transformation, we investigated the T-strand accumulation of various size of T-DNA in two kinds of binary vectors (one copy vs. multi-copy) upon acetosyringone (AS) induction and explored ways to improve the efficiency of the large T-DNA fragment transfer in *Agrobacterium*-mediated rice transformation. By performing immuno-precipitation of VirD2-T-strands and quantitative real-time PCR assays, we monitored the accumulation of the T-strands in *Agrobacterium tumeficiens* after AS induction. We further demonstrated that extension of AS induction time highly significantly improved large-size T-DNA transfer to rice cells.

**Conclusions:**

Our data provide valuable information of the T-strand dynamics and its impact on large T-DNA transfer in monocots, and likely dicots as well.

## Background

*Agrobacterium tumefaciens*-mediated genetic transformation is a powerful technology used to produce genetically modified transgenic plants [1]. It has been widely and routinely used in a large number of important economic dicots, including soybeans, cotton, canola, potatoes, and tomatoes [2]. Most monocot species are not natural hosts and were considered recalcitrant to *Agrobacterium*-mediated transformation. However, in recent years, monocot transformation efficiencies were substantially improved, such as in rice [3–6], corn [7–9], sorghum [10, 11], sugarcane [12, 13], and turfgrasses [14, 15], by adjusting various factors that help efficient delivery and integration of transgenes into the plant genomes, and by improvement of plant regeneration.

*Agrobacterium* infects plants by transferring a well-defined DNA fragment, namely transferred DNA (T-DNA), from its tumor-inducing (Ti) plasmid to the plant cell genome [16]. The processing and transfer of T-DNA are controlled by the activity of the virulence (*vir*) genes. The *virA* gene encodes a membrane-bound kinase that perceives chemical signals, such as the phenolic compound, acetosyringone (AS), from wounded plant cells. Once sensing the signal, VirA phosphorylates itself and activates the *virG* gene product, which stimulates the transcription of other *vir* genes and itself [17, 18]. VirD1/VirD2 proteins function together to generate single-stranded (ss) T-DNA molecules (called T-strands) [19, 20], and a single VirD2 molecule is covalently linked to the 5′ end of the T-strand, forming a ssDNA-protein complex called the immature T-complex [1, 19, 21]. It is believed that the immature T-complex, along with a few other virulence proteins such as VirE2, VirE3, VirD5, and VirF, is exported into the plant cells through a VirB/D4 type IV secretion system [20, 22, 23]. In vitro study show that numerous VirE2, the non-sequence-specific ssDNA binding proteins, non-covalently coat the entire length of the T-strand and pack it into a telephone cord-like coiled structure [24], which is thought to protect the T-strand from the nuclease degradation during its journey to plant cell nucleus. Both VirD2 and VirE2 contain the nuclear localization signals (NLSs) to mediate the nuclear import of the T-strands [16].

A major progress in *Agrobacterium*-mediated plant transformation was the creation of the binary system, in which the “disarmed” (with lack of the T-DNA, particularly the oncogenes) Ti plasmid containing all the *vir* genes serves as a helper plasmid, and a smaller replicon, containing the T-DNA region to facilitate transgene manipulation, serves as the binary vector [25].

The routine size of a natural T-DNA in a wild-type Ti plasmid is 5–30 kb, which encodes the oncogenes and opine biosynthesis genes [2]. However, when transfer of a large fragment of DNA (such as up to 100 kb) is needed for multi-transgene trait stacking, the naturally occurring machineries for transferring T-DNA are inefficient and insufficient [26]. In the 1990s, a few laboratories reported the transfer of very large DNA molecules (approximately 100–200 kb) into plants using *Agrobacterium*-mediated transformation [27–29]. Hamilton et al. [26, 27] designed new binary bacterial artificial chromosome (BIBAC) vectors for transferring large T-DNA molecules from *Agrobacterium* to plants. The BIBAC vectors are single copy plasmids, and have features of both a BAC vector designed for cloning large DNA fragment in *E.coli* and a binary vector designed to facilitate *Agrobacterium*-mediated transformation [26]. A 30-kb yeast genomic DNA fragment and a 150-kb human genomic DNA fragment were individually inserted into a BIBAC vector and successfully introduced into tobacco plants [26, 27]. A similar approach was later applied to rice transformation and similar results were obtained [30]. In those cases, a helper plasmid that carries an additional copy of *virG*, *virE1*, and *virE2* each was required and the transformation efficiencies were very low.

While transformation efficiencies of “small” DNA fragments have been remarkably improved for major cereal crops in recent years [31], large size DNA fragment transfer remains a bottleneck for those crops. In this study, by performing immuno-precipitation with anti-VirD2 antibodies coupled with qPCR, we measured accumulation of various sizes of T-strands from BIBAC2 and pCAMBIA1301 binary vectors in an attempt to use the information to improve the efficiency of large fragment transfer in the *Agrobacterium*-mediated transformation of rice, a model plant of cereal crops.

## Results

### Construction of binary vectors

**Fig. 1:**
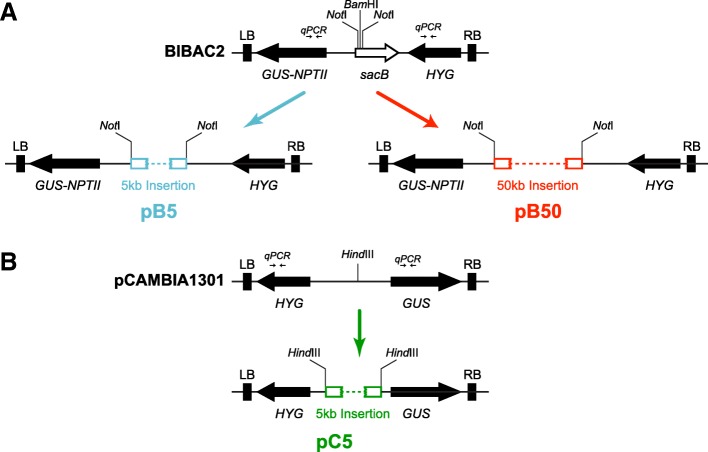
Plasmid constructs used in this study. **a** The scheme of the T-DNA regions of the BIBAC2 vector and BIBAC2 test constructs containing a 5-kb *Arabidopsis* genomic DNA fragment (pB5) or a 50-kb yeast genomic DNA fragment (pB50); **b** The scheme of the T-DNA regions of pCAMBIA1301 vector and pCAMBIA1301 test construct containing a 5-kb *Arabidopsis* genomic DNA fragment (pC5). “qPCR” indicates the locations of gene fragments of *GUS/HYG* that are amplified for qPCR assays

The binary vector BIBAC2 [27] is a single-copy plasmid that harbors a *GUS::NPTII* fusion gene and a hygromycin B resistant gene (*HYG*) located near the left and right border of the T-DNA, respectively. To examine the relationship between the T-DNA size of BIBAC2 and the transformation efficiency, two recombinant binary vectors were constructed. A random piece of 50-kb yeast genomic DNA and a 5-kb *Arabidopsis* genomic DNA fragment were individually cloned into BIBAC2, and the resulting recombinant binary vectors were named pB50 and pB5, respectively (Fig. 1a). In addition, the same 5-kb *Arabidopsis* genomic DNA was cloned into a multi-copy (10–20 per cell) binary vector pCAMBIA1301 [32, 33] with *HYG* and *GUS* gene located near the left and right border of the T-DNA, respectively, and the resulting vector was named pC5 (Fig. 1b). Comparison between pC5 and pB5 could indicate whether and how the copy number of the binary vector affects transformation efficiency. pB50, pB5 and pC5 were individually introduced into *Agrobacterium* strain AGL1, and the resultant strains are named B50, B5 and C5, respectively.

### T-strand formation and accumulation in *Agrobacterium* cells is affected by T-DNA size and the copy number of the binary vector

T-strand formation in *Agrobacterium* cells is a critical step in *Agrobacterium*-mediated plant transformation. To examine the factors that have impact on the T-strand formation in *Agrobacterium* cells, we performed T-strand immuno-precipitation (IP) using the anti-VirD2 antibody and qPCR assay to measure T-strands formation inside the *Agrobacterium* cells harboring pB50, pB5, or pC5 following AS induction.

**Fig. 2:**
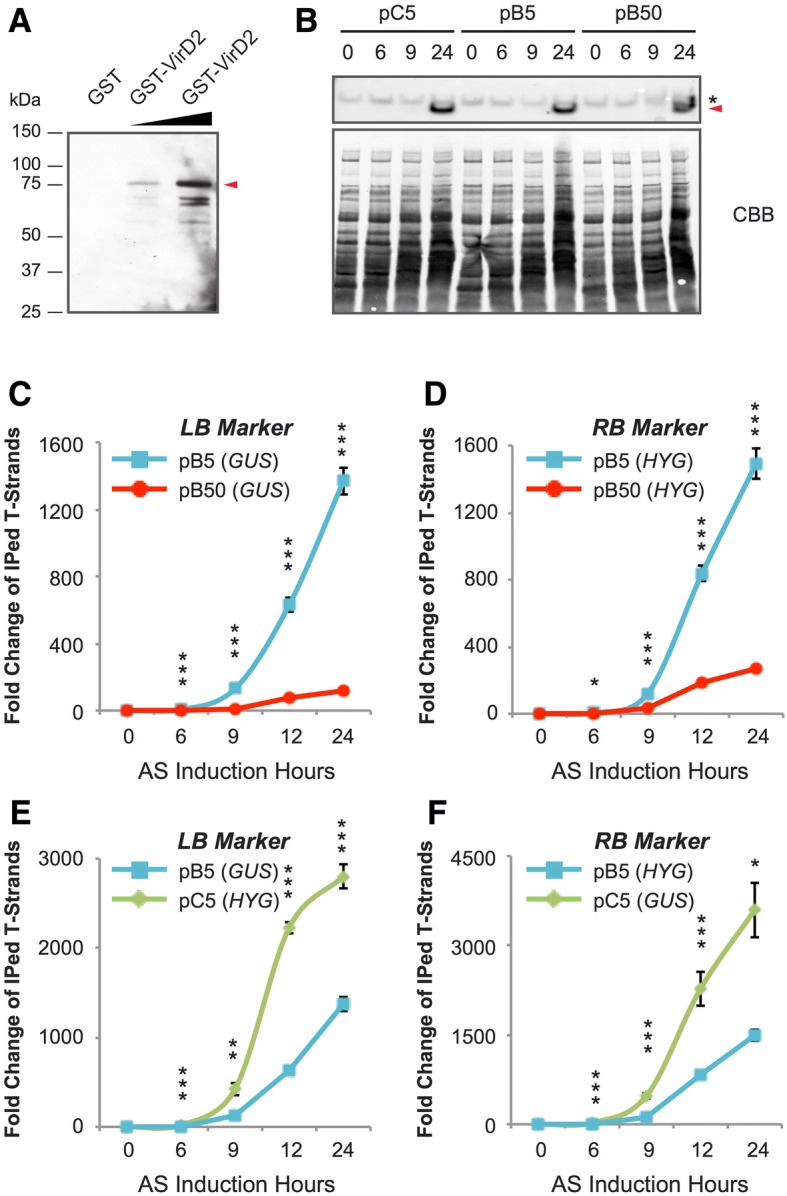
T-strand-immunoprecipitation and qPCR assay in *Agrobacterium* cells after AS induction. **a** Immunoblot analysis to examine the antibody against VirD2. The GST-VirD2, but not GST, can be detected by the antibody against VirD2. GST-VirD2 bands are indicated by the red arrow. Increased amount of GST-VirD2 (2.5 ng and 10 ng) is indicated by the black triangle. **b** Immunoblot analysis of VirD2 expression level (upper panel) in *Agrobacterium* cells after various hours of AS induction. Total protein is detected with Coomassie brilliant blue (CBB) staining (lower panel). **c** Comparison of the fold change of immunoprecipitated (IPed) T-strands between B5 and B50 tested with the left border marker. **d** Comparison of the fold change of IPed T-strands between B5 and B50 tested with the right border marker. **e** Comparison of the fold change of IPed T-strands between C5 and B5 tested with the left border marker. **f** Comparison of the fold change of IPed T-strands between pC5 and pB5 tested with the right border marker. Data represent means and standard errors (*n* = 3). ***p < 0.001, **p < 0.01, *p < 0.05 show significance level in Student’s t test

We first confirmed the functionality of the anti-VirD2 antibody. As shown in Fig. 2a, the anti-VirD2 antibody was able to detect GST-VirD2 fusion protein but not GST only. Next, we examined the expression level of VirD2 after AS induction. Samples with similar number of bacterial cells were collected at 0, 6, 9, and 24 h after AS induction. The expression of VirD2 was hardly detected by 9 h but became very obvious at 24 h after AS induction (Fig. 2b).

Excessive amount of the anti-VirD2 antibody was used in T-strand-IP in order to maximize immature T-complex binding and capture. The fold increases of the T-strands were quantified by qPCR by measuring the two transgenes near the right and left border of the T-DNA (*GUS* and *HYG*), respectively, and normalized against *Agrobacterium* chromosomal marker gene *dnaK*. Our data showed that the amount of T-strands was highly significantly increased after AS induction within 24 h in all three strains of *Agrobacterium* cells (Fig. 2c-f). As shown in the figures, at 6 h, significant differences were already seen between B5 and B50 as well as between C5 and B5. From 9 to 12 h, the amount of T strands in B5 and C5 increased near-exponentially while fold increase in B50 just started. From 12 to 24 h, the increase rates in C5 and B50 were down slightly whereas B5 still maintained exponential-like fold increase. By 24 h, the amount of T-strands in C5 strain increased by around 3000 fold, while the accumulation in B5 and B50 strains rose to ~ 1500 and 100–250 fold, respectively. The results showed that the amount of T-strands increased remarkably upon AS induction in all three strains, and the increase was negatively impacted by the T-DNA size and positively affected by the copy number of the binary vector (Fig. 2c-f). The results also demonstrated that longer AS induction (such as 24 h) was beneficial to T-strand formation and accumulation, and thus might facilitate improvement of transformation efficiency.

### Longer AS induction improves large DNA fragment transfer

**Fig. 3:**
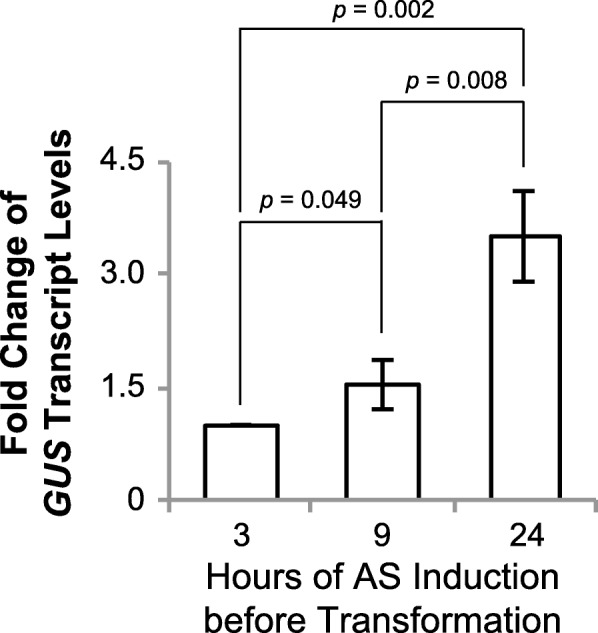
The effect of AS induction time on *GUS* transcript level in B50 infected rice calli. The real-time qPCR analysis shows that the AS induction time (3, 9 and 24 h) affects the *GUS* transcript level in infected rice calli. Rice transformation was performed as described above. Samples of infected rice calli were collected after 3 days of co-cultivation. Total RNA was extracted and qRT-PCR was performed for transient GUS transcripts. The relative fold change of *GUS* expression level is calculated against the *GUS* expression level with 3-h AS induction before transformation. Data represent means and standard errors (*n* = 3). The *p* values in Student’s t tests are shown

AS is a naturally occurring phenolic compound upon plant wounding, and induces expression of *vir* genes in *Agrobacterium* [18, 34]. It is a common practice to add AS in bacterial culture to activate the *Agrobacterium* virulence for monocot transformation [4]. However, the literature is ambiguous with regards to an optimal AS treatment duration. Our results indicate that the longer duration (24 h) of the treatment leads to significantly higher accumulation of the T-strands in the *Agrobacterium* cells (Fig. 2c-f). We hypothesize that longer AS induction may improve the transformation efficiency, particularly for the single-copy vectors and the larger size T-DNAs since longer time of AS induction produced hundreds of fold more T-strands (Fig. 2c, d). To validate this hypothesis, AS-treated B50 strain collected at three different time points (3, 9 and 24 h) was used to transform rice calli, and the transiently-expressed *GUS* transcripts were quantified by qRT-PCR following 3-day co-cultivation. Our data clearly showed that by 24 h, the *GUS* transcript level increased by ~ 3.5 fold from 3 h, which was highly significant when compared to 3 and 9 h (*p* = 0.002 and 0.008, respectively, Fig. 3), an indicator of the improved efficiency of transient transformation, suggesting that extended AS induction time could be a valuable approach to improve large size T-DNA transfer to rice cells.

## Discussion

In *Agrobacterium*-mediated genetic transformation, transferring genes of interest into plants followed by stable integration and transgene expression are influenced by multiple factors, such as the genotype and activation status of *Agrobacterium* strain, host plant genotype and cell status, physical treatments, and plant defense reactions to *Agrobacterium* infection [2, 5, 7, 8, 15, 31, 35]. There is an increased demand in transferring large DNA fragments containing multiple transgenes, but the transformation efficiencies are very low in both dicots [27] and monocots [30]. The reasons of this phenomenon are poorly understood. In this research, we employed a novel approach of T-strand-virD2 immuno-precipitation coupled with qPCR to monitor the accumulation of immature T-complex in AS-treated *Agrobacterium* and gained more insights of the T-strands formation process. We then used the gained knowledge to guide our approaches for improved transformation efficiency. To evaluate the transformation affected by the copy number of the binary vectors, we compared T-strands accumulation between strains B5 and C5, representing single or multiple copies of the binary vectors, respectively. We observed that, by 24 h of AS treatment, the total amount of T-strands (as measured from RB gene) in C5 increased by ~ 3000-fold whereas the increase in B5 was only half of the value (Fig. 2f). The fold increase indicates that the copy number of the binary vector is positively correlated with the T-strand accumulation in the infected rice cells. The result is in alignment with Zhi et al. [36], who reported higher transformation efficiency with multiple plasmid copy number in maize. It has been reported that the copy number of the wild-type Ti plasmid increases moderately from 1 to 5 per chromosome within 24 h of AS induction compared with that in the non-induced cells [17, 37], and T-strand number is moderately increased by 12–14 fold per Ti plasmid within the same period [37], indicating that the total T-strand number increase could be attributed to both replication of Ti plasmid and generation of T-strands upon AS induction. Little is known on the process of the T-strand generation. It is believed that T-strand is released by strand-replacement synthesis from the Ti plasmid [38]. All the previous reports of T-strand increase were on *Agrobacterium* containing the wild-type Ti plasmids. In our experiments, we investigated T-strand accumulation from the “man-made” binary vectors, and observed more robust T-strand generation from these vectors than from the wild type Ti plasmids. The mechanism of this phenomenon remains to be elucidated.

Our data do show that longer T-DNA (like 50 kb in pB50) reduced the T-strands formation by 5–7 fold (24 h after AS induction) as compared to pB5 (5 kb, Fig. 2c, d). Based on our observations on T-strand formation, we found that longer AS induction duration could remarkably enhance T-strands accumulation (Fig. 2c-f). We hypothesized that accumulation of higher number of T-strands could facilitate T-strand transfer to plant cells during infection process, which would lead to improved large T-DNA transformation. In that direction, we compared 3, 9 and 24 h of AS induction time, quantified *GUS* reporter gene transcripts, and observed highly significant fold increase of *GUS* transcripts in the infected rice cells (Fig. 3). Since *GUS* gene in this construct is near the left border of the T-DNA, it is reasonable to assume that the result reflects full-length or near full-length of the functional T-DNAs. In this experiment, the longest period of AS treatment tested was 24 h, which had the highest *GUS* transcript accumulation. It is likely that 24-h AS treatment is still not the optimized duration, but our results reveal a new research direction. Longer induction durations and other possible factors affecting *Agrobacterium* activation status and/or elevating synthesis of the long T-strands need to be tested in future experiments. Moreover, a comparison of transient expression of the transgenes and stable transformation would help gain more insights on the T-strand transfer.

Figure 2 demonstrates that it takes time for virD2 gene expression. In Fig. 2c-f, we reported increase of immuno-precipitated T-strands starting at 6 and 9 h of AS treatment, but the corresponding increase of virD2 formation could not be clearly told in the immunoblot (Fig. 2b). We believe that the discrepancy is caused by the sensitivity level of the two research methods. The qPCR used in Fig. 2c-f is way more sensitive than the immunoblot, which depends on antigen-antibody recognition, enzyme reaction, and film exposure. qPCR is a quantitative method while the immunoblot is a more qualitative, at most a semi-quantitative, method which we often cannot tell the subtle differences, especially at an early stage of the induced gene expression.

## Conclusion

By performing virD2 immuno-precipitation, we monitored a time course of the T-strand accumulation in *Agrobacterium* upon AS induction, and demonstrated that larger T-DNA size reduced the number of T-strand accumulation, which could be a main cause of the low transformation efficiency. Increasing T-strand number by extension of AS induction period could be a promising approach to significantly improve large size T-DNA transfer efficiency in rice transformation. With this simple approach, we assume similar positive results could be expected in many other plant species, monocots or dicots. In addition, based on the data collected in these experiments, we anticipate that the extended AS treatment approach could help improve small-size T-DNA transformation as well.

## Methods

### Constructs, bacterial strain, antibodies and recombinant proteins

A random piece of 50-kb yeast genomic DNA was isolated using CHEF Yeast Genomic DNA Plug Kit (Cat#170–3593, Bio-Rad, Hercules, CA) and a 5 kb *Arabidopsis* genomic DNA fragment was individually cloned into BIBAC2 between the two *Not*I sites as single copy binary vectors pB50 and pB5, respectively. The same 5 kb *Arabidopsis* genomic DNA fragment was cloned into pCAMBIA1301 at the *Hin*dIII site to serve as a multi-copy binary vector pC5. Each binary vector was introduced into *Agrobacterium* strain AGL1 individually. AGL1 is a *recA*-deficient derivative of *A. tumefaciens* strain C58 [39] and was acquired from Dr. Lynn Dahleen, USDA, ARS, Fargo, ND. Binary vectors BIBAC2 and pCAMBIA1301 were acquired from Cornell University, Center for Technology Enterprise and Commercialization (CCTEC), and CAMBIA (Canberra, Australia), respectively. The antibody against VirD2 was kindly provided by Dr. Zambryski at UC-Berkeley [40] and Dr. C. Baron at the University of Montreal (Montreal, Canada).

For expression and purification of the recombinant protein, a full-length gene of *virD2* (amplified with primers *virD2*Fwd: 5’-GGGTCCATGGATATCGGGATGCCCG ATCGCGCTCA AG-3′, and *virD2*Rev: 5’-TGCTCGAGTGCGGCCGCACTAGGTC CCCCCGCGCC CA-3′) was cloned into the bacterial expression vector pET42b (Novogen, Hornsby Westfield, Australia) between *Bam*HI and *Hin*dIII using Gibson assembly (New England Biolabs, Ipswich, MA). 6xHis-tagged GST fusion protein was expressed in *Escherichia coli* strain BL21 (DE3) (Agilent Technologies, Santa Clara, CA). The cell pellets were lysed by French press in the lysis buffer containing 50 mM NaH_2_PO_4_, 300 mM NaCl, 10 mM imidazole, and protease inhibitor cocktail (Sigma-Aldrich, St. Louis, MO). The cell extract was prepared by centrifugation at 20,000 *g* for 20 min at 4 °C. The cleared cell extract was incubated with His60 Ni Superflow Resin (Clontech, Mountain View, CA) equilibrated in the lysis buffer at 4 °C for 2 h. The recombinant proteins were subsequently purified in the gravity-flow column following the manufacturer’s instructions (Clontech).

### Sample preparation

For *Agrobacterium* samples, *Agrobacterium* strain AGL1 harboring various binary vectors was grown overnight in 150 ml YEP medium containing 25 mg/L rifampicin and 50 mg/L kanamycin at 28 °C. Cells were then centrifuged and re-suspended in 150 ml AB induction medium [41]. The OD_600_ reading of the culture was adjusted to 0.6, and six ml from each culture was collected at this point as non-induced samples. Then acetosyringone (AS) was added (final concentration 200 μM) to each of the remaining cultures. Cultures were grown at room temperature (RT). *Agrobacterium* samples were collected at 6, 9, 12 and 24 h after AS addition, immediately washed by 20 mM sodium phosphate buffer (pH 6.8) and pelleted for lysate extraction. When sampling, the OD_600_ value of each sample was measured and adjusted to 0.6.

Lysate extraction from *Agrobacterium* cells was prepared after sampling [42]. Briefly, cells were re-suspended in 200 μl TES buffer (50 mM Tris-HCl pH 6.8; 2 mM EDTA; 1% β-mercaptoethanol; 1% SDS) and shaken for 30 min at 37 °C. Then, 900 μl of NP1 buffer (150 mM Tris-HCl pH 8.0, 0.5 M sucrose, 10 mM EDTA) containing 1 mg/ml lysozyme was added. The mixture was incubated on ice for at least 1 h, followed by 30 min shaking at 37 °*C. triton* X-100 was then added to a 4% final concentration and the mixture was incubated for 15 min at RT. Three hundred and forty-three μl of 5× EDTA-free protease inhibitors cocktail (in 25 mM MgCl_2_, Roche Applied Science, Penzberg, Germany) was added and the mixture was rotated for 15 min at 37 °C followed by 2–3 h at 4 °C. The insoluble material was removed by centrifugation at 18,400 g for 15 min and the supernatant was collected. The protein concentration of the total soluble lysate was determined by the Bradford reagent (Bio-Rad) and diluted to a final concentration of 0.5 mg/ml. One mL of the diluted lysate was used for T-strand immune-precipitation (T-strand-IP). Another 100 μL of the same diluted lysate was treated with DNase-free RNase (Thermo Fisher Scientific, Waltham, MA), Proteinase K (Thermo Fisher Scientific) and then precipitated by ethanol to obtain the genomic DNA, which would be used as the “input” in the T-strand-IP and qPCR assays described later.

For rice calli samples, rice (*Oryza sativa* L.) cultivar ‘Taipei 309″ seeds were used for callus induction as previously described [5]. The calli were sub-cultured every two weeks. Two- to three-month-old healthy friable rice calli were selected for *Agrobacterium* transformation. *Agrobacterium* culture was used to infect 100–120 pieces of rice calli according to Patel et al. [5]. Infected calli were blotted onto three layers of sterile filter paper to remove the excessive *Agrobacterium* suspension and then put on clean sterile filter papers for sample collection. Healthy rice calli without *Agrobacterium* infection were used as controls.

### Immunoblot analysis

Concentration of the total soluble proteins was determined by the Bradford reagent (Bio-Rad). Equal amounts of total proteins (20 μg) from each sample were subjected to SDS-PAGE and subsequently transferred to polyvinylidene fluoride (PVDF) membrane (Millipore, Billerica, MA). After blocking with 2% non-fat milk in TBST buffer (50 mM Tris-HCl, pH 7.5; 150 mM NaCl; 0.05% Tween-20), the membrane was incubated in the same buffer containing 1/1000 dilution of rabbit anti-VirD2 antibodies. After three times of washing with TBST buffer, the membrane was incubated with 2% non-fat milk in TBST buffer containing 1/5000 dilution of the goat anti-rabbit HRP (Thermo Fisher Scientific). The signal was detected with the SuperSignal West Pico Chemiluminescent Substrate (Thermo Fisher Scientific).

### T-strand-immunoprecipitation and qPCR assay of *Agrobacterium* cells

One mL of diluted lysates (0.5 mg/mL) extracted from *Agrobacterium* cells were incubated with the antibodies against VirD2 at 4 °C overnight. Meanwhile, the Dynabeads™ Protein G magnetic beads (Thermo Fisher Scientific) were blocked with 20 μg BSA (New England BioLabs) and 20 μg glycogen (Thermo Fisher Scientific) at 4 °C overnight. The next day, the magnetic beads were washed twice using the extraction buffer NP1, and then re-suspended in appropriate volume of NP1 buffer. Forty μL of equilibrated magnetic beads were then added to the mixture of lysate and antibody against VirD2, and the total mixture was incubated for four hours at 4 °C. After washing with NP1 buffer for four times, freshly prepared elution buffer (1% SDS, 0.168 g NaHCO_3_/20 ml buffer) was used to elute the products of immuno-precipitation at 65 °C. The eluate was digested by Proteinase K at 55 °C overnight and then purified using phenol-chloroform extraction. The final products (T-strand DNA) were precipitated by ethanol.

qPCRs were performed with iTaq Universal SYBR Green Supermix (Bio-Rad) to analyze T-strand formation in *Agrobacterium* cells on a Real-Time PCR Instrument (Agilent). *Agrobacterium* chromosome marker *dnaK* (with primers of *dnaK*Fwd: 5’-TACCTTCCTCGGTGGTGAAG-3′, and *dnaK*Rev: 5’-CGAGGACGAAAGTTC GATC-3′) was amplified from the 100 μL diluted lysate prepared previously and used as an internal control. T-strand marker gene *HYG* (*HYG*Fwd: 5’-GGTCGCCAACATCTT CTTCT-3′, *HYG*Rev: 5’-CGAAATTGCCGTCAACCAAG-3′) or *GUS* (*Gus*Fwd: 5’-ACGTCTGGTATCAGCGCGAAGTC-3′, *Gus*Rev: 5’-TATAGCCGCCCTGATGCTCC ATC-3′) was amplified from the purified T-strand-IP products. Relative quantification using comparative CT calculation method was applied in qPCR data analysis.

### Transient *GUS* transcripts analysis in the infected calli

*Agrobacterium* cells harboring the binary vector of pB50 were grown overnight in 15 ml of YEP medium containing 25 mg/L rifampicin and 50 mg/L kanamycin at 28 °C. The next day, the overnight culture was divided into 3 equal aliquots (5 ml each). Forty-five ml of fresh infection medium containing 200 μM AS was added to each aliquot for induction at RT. The cultures were sampled at three time points (3, 9, and 24 h). The OD_600_ of each 50 ml *Agrobacterium* suspension was examined and adjusted to 0.6 before rice calli infection. The rice transformation was performed as previously described [5]. After a three-day co-cultivation, samples were collected, washed thoroughly with 200 mg/L timentin (GlaxoSmithKline, Research Triangle Park, NC) and stored at − 80 °C.

Total RNA was isolated from the infected rice calli using the Quick-RNA MicroPrep (Zymo Research, Irvine, CA), and then treated with DNase I (RNase-free DNase set; Zymo Research) to clean up potential contamination of the genomic DNA. Concentration of the total RNA was quantified using a NanoDrop 2000C spectrophotometer. The first-strand cDNAs were synthesized using iScript cDNA Synthesis Kit (Bio-Rad). Subsequent real-time RT-PCRs were performed with iTaq Universal SYBR Green Supermix (Bio-Rad) to analyze *GUS* transcripts on a Real-Time PCR Instrument (Agilent). The housekeeping gene *UBQ5* was used as an internal control [43]. The relative fold change of *GUS* transcripts is calculated against the *GUS* expression level with 3-h AS induction before transformation.

### Statistical analysis

Two-tailed student t-test was carried out to evaluate significant differences in values of qPCR and qRT-PCR. “*” indicates significant differences: ***p < 0.001, **p < 0.01, *p < 0.05. Data presented are means ± standard errors (*n* = 3).

## Abbreviations

AS: Acetosyringone
GUS: β-glucuronidase
HYG: Hygromycin B resistance gene
IP: Immuno-precipitation
LB: Left border
NLS: Nuclear localization signal
qPCR: Quantitative polymerase chain reaction
qRT-PCR: Quantitative reverse transcription polymerase chain reaction
RB: Right border
T-DNA: Transferred DNA
